# Dipolar elementary current systems for ionospheric current reconstruction at low and middle latitudes

**DOI:** 10.1186/s40623-020-01284-1

**Published:** 2020-10-15

**Authors:** Heikki Vanhamäki, Astrid Maute, Patrick Alken, Huixin Liu

**Affiliations:** 1 Ionospheric Research Unit, University of Oulu, Oulu, Finland; 2 High Altitude Observatory, National Center for Atmospheric Research, Boulder, Colorado USA; 3 Cooperative Institute for Research in Environmental Sciences, University of Colorado, Boulder, Colorado USA; 4 Department of Earth and Planetary Sciences, Kyushu University, Fukuoka, Japan

**Keywords:** SECS method, Ionospheric current, Magnetic field

## Abstract

The technique of spherical elementary current systems (SECS) is a powerful way to determine ionospheric and field-aligned currents (FAC) from magnetic field measurements made by low-Earth-orbiting satellites, possibly in combination with magnetometer arrays on the ground. The SECS method consists of two sets of basis functions for the ionospheric currents: divergence-free (DF) and curl-free (CF) components, which produce poloidal and toroidal magnetic fields, respectively. The original CF SECS are only applicable at high latitudes, as they build on the assumption that the FAC flow radially into or out of the ionosphere. The FAC at low and middle latitudes are far from radial, which renders the method inapplicable at these latitudes. In this study, we modify the original CF SECS by including FAC that flow along dipolar field lines. This allows the method to be applied at all latitudes. We name this method dipolar elementary current systems (DECS). Application of the DECS to synthetic data, as well as Swarm satellite measurements are carried out, demonstrating the good performance of this method, and its applicability to studies of ionospheric current systems at low and middle latitudes. 
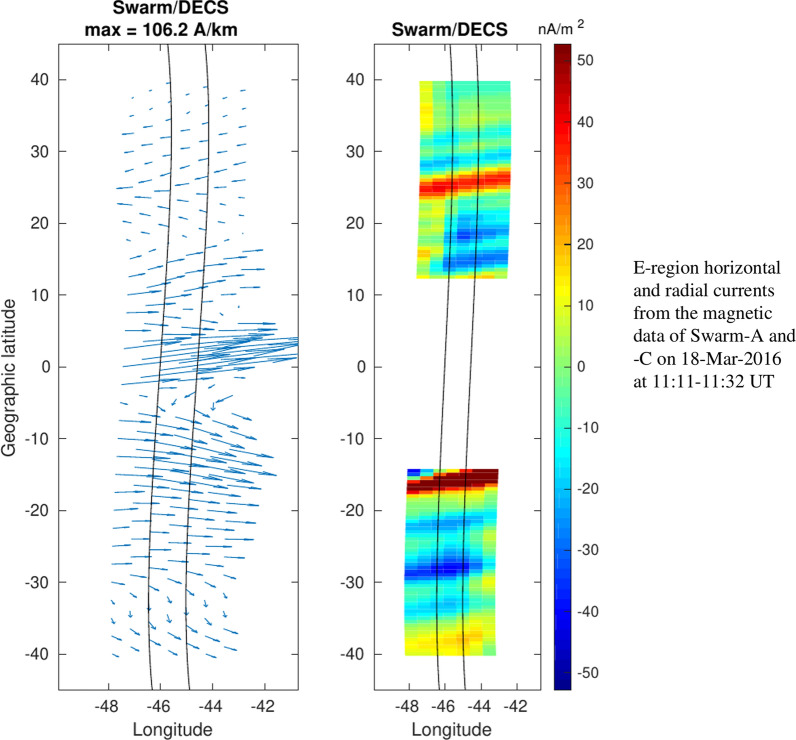

## Introduction

At middle and low magnetic latitudes the main driver of ionospheric currents is the neutral wind and the collisional interaction between the charged and neutral particles. The neutral wind field itself is driven by solar heating and solar and lunar atmospheric tides. A comprehensive review of the middle and low-latitude current systems is given by Yamazaki and Maute (2016). The overall large-scale current system forms two oppositely directed vortices at the northern and southern hemisphere on the dayside. This is named solar-quiet (Sq) current system, as it shows a strong dependence on solar local time and is present even during geomagnetically quiet conditions.

Two prominent current systems considered in this work are the interhemispheric field-aligned currents (IHFAC) and the equatorial electrojet (EEJ). The neutral wind field and electric conductivity may be different in the northern and southern hemispheres (e.g., due to seasonal effects), so there may be differences in the electric current and electric potential in the two hemispheres. This results in IHFAC flowing along the magnetic field lines between the Sq current systems at the two hemispheres (e.g., Fukushima 1979; Park et al. 2011). Due to the almost horizontal magnetic field and the generation of polarization electric field through the Cowling mechanism, the electric current is greatly enhanced in a relatively narrow strip centered at the magnetic dip equator (e.g., Forbes 1981; Lühr et al. 2004), creating the EEJ.

Magnetic measurements, either by ground-based arrays or by low-Earth-orbiting (LEO) spacecraft, are the main data source for probing ionospheric currents (e.g., Olsen 1997; Takeda 2002; Yamashita and Iyemori 2002; Lühr et al. 2004; Yamazaki et al. 2010; Park et al. 2011). Close to the magnetic equator the IHFAC are almost horizontal, but poleward of about $\pm 10^\circ$ magnetic latitude it is useful the separate the currents into horizontal sheet currents flowing in the ionospheric E-region and the IHFAC flowing along the magnetic field lines. Curl-free sheet currents (mostly Pedersen current) associated with the IHFAC make up current circuits in the meridional plane of the ionosphere, while the divergence-free sheet currents (mostly Hall current) form closed loops in the ionospheric plane. To unravel the spatial structure of the Sq current system, we need to take into account not only the equivalent currents estimated from ground magnetic measurements, but also the IHFAC and associated curl-free currents best estimated from satellites. The multi-satellite Swarm mission (Olsen et al. 2013) has opened up new possibilities for studying the Sq and EEJ (e.g., Chulliat et al. 2016; Alken et al. 2017) as well as the IHFAC (e.g., Lühr et al. 2015), as simultaneous measurements from multiple satellites remove many limitations of the previous single-satellite missions.

The method of spherical elementary current systems (SECS) introduced by Amm (1997) has proven to be a powerful tool for studying the high-latitude current systems. Vanhamäki and Juusola (2020) give a comprehensive review of the SECS method in general, while Amm et al. (2015) and Vanhamäki et al. (2020) discuss application of the SECS method to magnetic data provided by the parallel flying Swarm-A and -C satellites (Swarm/SECS). The main advantage of the Swarm/SECS method is that it is able to produce 2-dimensional (2D) latitude–longitude maps of the currents in a limited region around the satellites’ ionospheric footpoints.

The SECS method involves a simplifying assumption of radial FAC. Although this assumption is nearly satisfied at high latitudes, it is not valid at low and middle latitudes where the FAC are far from radial. In this study, we modify the SECS method by reformulating the relevant basis functions so that the FAC flow along dipolar field lines. This makes the method applicable to all latitudes, apart from a narrow strip around the magnetic equator, where the IHFAC are almost horizontal and thus a separation into horizontal sheet currents and IHFAC is not meaningful (see e.g., section 7 in Richmond 1995). We name this modified method dipole elementary current systems (DECS).

## Theory

The SECS consists of divergence-free (DF) and curl-free (CF) basis functions, which represent the height-integrated horizontal currents assumed to flow in a thin spherical shell at the ionospheric E-region. The DF systems are rotational, while the CF systems are divergent currents associated with FAC. Amm (1997) and Amm and Viljanen (1999) introduced the 2-dimensional (2D) SECS method, which can be used to estimate equivalent ionospheric currents from ground network observations (Amm and Viljanen 1999) or the actual currents from satellite measurements (Juusola et al. 2014; Amm et al. 2015). In order to use observations from a meridional chain of ground magnetometers or single satellite pass, the 1-dimensional (1D) SECS were introduced by Vanhamäki et al. (2003) and Juusola et al. (2006).

The 1D and 2D-CF SECS include assumption of radial FAC, so they need to be modified for low-latitude applications. In this section we introduce the 1D and 2D-CF DECS, where the FAC flows along dipole field lines, and calculate their magnetic field perturbations. In contrast, the 1D and 2D-DF SECS represent currents that are closed in the ionosphere and are not connected to the FAC, so they can be used at all latitudes. Relevant formulas for the current density of the 1D and 2D-DF SECS (which we interchangeably will also call the DF DECS) can be found for example in Vanhamäki and Juusola (2020), so they will not be discussed further here.

### 1D-CF DECS

The 1D-CF SECS have been modified to dipole geometry by Deguchi et al. (2013). The idea is to place two oppositely directed 1D-CF SECS at co-latitudes $\theta _0$ and $\pi - \theta _0$ in the dipole-oriented coordinate system. Using the expression of the 1D-CF SECS (Equation 2.37 in Vanhamäki and Juusola 2020) and assuming $\theta _0 < \pi /2$, the horizontal current density is

1 $$\begin{aligned} \vec {J}^{ 1DCF }_D(\theta ,\theta _0) = \frac{I_0}{R_I} {\vec {e}_{\theta }} \left\{{\begin{array}{*{20}{c}}{\sin \theta }&{{\theta _0} < \theta < \pi - {\theta _0}} \\0&{{\text{otherwise}}}\end{array}} \right. \end{aligned}$$

**Fig. 1:**
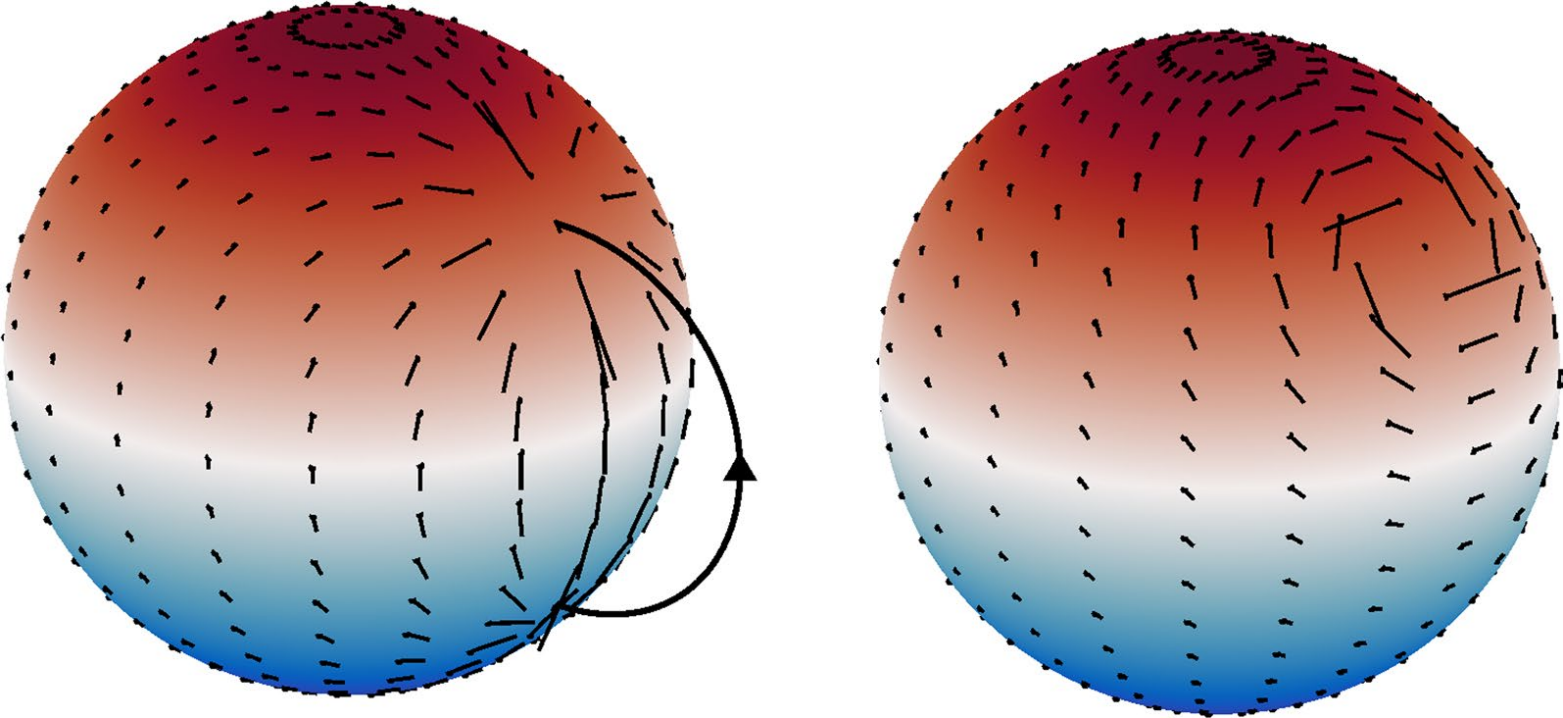
The current density in the 2D-CF DECS on the left and 2D-DF SECS/DECS on the right. Color represents dipole latitude. In the illustrated case $\theta _0 = 55^\circ$

Here, $R_I$ is the radius of the ionospheric current sheet (typically about 110 km altitude) and $\vec {e}_{\theta }$ is a unit vector in the southward direction. This kind of horizontal current has two rings of oppositely directed Dirac $\delta$-function divergences at co-latitudes $\theta _0$ and $\pi - \theta _0$, with zero divergence elsewhere. The divergences at opposite hemispheres are connected by FAC flowing along dipole field lines. For illustration see the left panel in Fig. 1, but imagine that the illustrated current systems are placed at all longitudes (i.e., the current has no longitudinal gradients).

The magnetic field of the 1D-CF DECS can be calculated using symmetry arguments and Ampere’s law, as in Appendix A and B of Juusola et al. (2006). The result is

2 $$\begin{aligned} \vec {B}_D^{ 1DCF }(r,\theta ,\theta _0) = - \mu _0 \left( \frac{R_I}{r} \right) ^\frac{3}{2} \left\{ \begin{array}{ll} \vec {e}_{r} \times \vec {J}^{ 1DCF }_D(\theta _I,\theta _0), &\,r>R_I \\ 0, &\,r<R_I \end{array} \right. , \end{aligned}$$

where $\vec {e}_{r}$ is the radial unit vector. In a dipole magnetic field the magnetic footpoint of a point $(r,\theta )$ is at co-latitude

3 $$\begin{aligned} \sin \theta _I = \sqrt{\frac{R_I}{r}} \sin \theta . \end{aligned}$$

These kind of basis function impose strict anti-symmetry for the FAC between the northern and southern hemispheres. While the IHFAC at middle and low latitudes should indeed flow from one hemisphere to the other, we can not expect a strict anti-symmetry to be valid. Therefore, in practical applications we should consider analyzing data from the two hemispheres separately.

It should be noted that together the dipolar FAC and horizontal current in Eq. (1) form a closed toroidal current loop around the Earth, with the magnetic field confined inside the loop. This kind of current system is totally invisible to ground-based magnetometers and spacecraft can detect it only if they orbit so low that they intersect the dipolar FAC. Even LEO spacecraft cannot detect the IHFAC arising from a small region around the magnetic equator, where the field lines do not reach the orbital altitude.

### 2D-CF DECS

We obtain a 2D-CF DECS by placing two oppositely directed 2D-CF SECS at conjugate points $(\theta _0,\phi _0)$ and $(\pi -\theta _0,\phi _0)$ in the ionosphere and connect them by FAC flowing along the dipole field from one hemisphere to the other. The ionospheric horizontal current of this dipolar 2D-CF DECS can be written as

4 $$\begin{aligned} \vec {J}_D^{ 2DCF } = \vec {J}^{ 2DCF }(north) - \vec {J}^{ 2DCF }(south), \end{aligned}$$

where $\vec {J}^{ 2DCF }$ is the horizontal current of a 2D-CF SECS (given in Eq. 2.7 of Vanhamäki and Juusola 2020). This current system is illustrated in Fig. 1, together with the 2D-DF elementary system. Note that divergence of the horizontal current in Eq. (4) is zero, except for two $\delta$-functions at $(\theta _0,\phi _0)$ and $(\pi -\theta _0,\phi _0)$.

The magnetic field of the modified dipolar 2D-CF SECS system is calculated in the Appendix. It can be written symbolically as

5 $$\begin{aligned} \vec {B}_D^{ 2DCF } = \vec {B}_{1}(north) + \vec {B}_{2}(north \rightarrow south) - \vec {B}_{1}(south), \end{aligned}$$

where $\vec {B}_{1}(north) - \vec {B}_{1}(south)$ is the magnetic field of the horizontal currents, while $\vec {B}_{2}$ is the field created by the dipolar FAC. Expressions for these parts are given in Eqs. (11) and (15).

We need to calculate the magnetic field along the orbit of the Swarm satellites. The calculation point will often be very close to the idealized line current flowing along the dipole. This brings numerical problems, because the Biot–Savart formula is proportional to $distance^{-3}$. However, we have found that a reasonably accurate and numerically stable approximation can be achieved by simply imposing a lower limit to the distance used in the numerical integration. Basically we evaluate the sum in Eq. (15) with the replacement

6 $$\begin{aligned} |\vec {r}_{D,i}-\vec {r}| \rightarrow max(|\vec {r}_{D,i}-\vec {r}|,L_{min}), \end{aligned}$$

where the minimum accepted distance $L_{min}$ is selected as half of the distance between the DECS poles in the ionospheric analysis grid. Additionally, whenever we need to evaluate the integral close to the line current, specifically whenever the distance between the DECS pole and the ionospheric footpoint of the calculation point is smaller than $2.5 L_{min}$, we sub-divide the 2D-CF DECS into 9 sub-poles arranged to a $3 \times$ latitude/longitude grid. Each sub-pole is then treated as having 1/9 part of the original 2D-CF DECS’s amplitude and a new minimum distance $L_{min}/3$.

### Swarm/DECS method at low and middle latitudes

A detailed description of the Swarm/SECS analysis method and its applications at high latitudes is given by Amm et al. (2015) and Vanhamäki et al. (2020). The Swarm/DECS analysis developed here is done in an analogous manner, except that the 1D and 2D-CF SECS are replaced by the 1D and 2D-CF DECS. For completeness sake, we describe the main analysis steps here.

The Swarm/DECS analysis takes as input the positions ($\vec {r}^{sat}$) and the magnetic measurements ($\vec {B}^{sat}$) of the Swarm-A and -C satellites. The Earth’s main field, lithospheric field and magnetospheric contributions need to be subtracted from the Swarm magnetic field data using suitable models, such as CHAOS (Finlay et al. 2016). Several 1D and 2D-CF and DF elementary systems are placed to a regular grid at the ionospheric E-region around the satellite paths. We use a similar grid as in Amm et al. (2015) and Vanhamäki et al. (2020), with spatial resolution of $0.5^\circ$ in latitude and half of the longitudinal spacing between the Swarm-A/C satellites in longitude. Output parameters are the ionospheric horizontal sheet current ($\vec {J}_\perp$) and IHFAC along a strip around the ionospheric projection of the satellite tracks.

The analysis proceeds by fitting 4 different current systems (1D/2D and CF/DF DECS) to the measured magnetic variation field one by one:

1. Fit 1D-DF SECS using only the magnetic variation field component parallel to the main field $B_\parallel$,
2. Fit 2D-DF SECS using residual $B_\parallel$,
3. Fit 1D-CF DECS to the residual $\phi$-component (eastward) of the magnetic field,
4. Fit 2D-CF DECS to the residual $B_r$, $B_\theta$ and $B_\phi$.

Ordering of the above analysis steps is a result of two factors. Firstly, the large-scale electrojet type current systems are fitted with 1D systems whenever possible, as the amount of input data is limited to 2 satellite tracks. Secondly, $B_\parallel$ is mostly produced by the divergence-free ionospheric currents, whereas the horizontal components are dominated by FAC connected to the curl-free ionospheric current.

In each of the above steps we have a matrix equation between the measured field components and the unknown amplitudes of the elementary systems. The matrix inversions are regularized with truncated singular value decomposition, where the selection of the truncation point is done by optimizing the result in synthetic test cases.

### Local dipole coordinates

In many areas around the globe the Earth’s magnetic field can be very different from an ideal dipole. Therefore, we will carry out the analysis in a local dipole coordinate system, which we define as a spherical coordinate system whose orientation is chosen so that a dipole field in that coordinate system matches the Earth’s magnetic at the measurement points (i.e., the Swarm-A/C orbits) as closely as possible.

The best orientation for the local dipole system is found by minimizing

7 $$\begin{aligned} S = < \left| \vec {e}_{D} - \vec {e}_{data} \right| ^2 >, \end{aligned}$$

where $<>$ means spatial average over the Swarm measurement points, $\vec {e}_{D}$ is a unit vector along a dipole field and $\vec {e}_{data}$ is a unit vector along the the Earth’s main field at the measurement points. Minimization is done for the difference of the unit vectors, because it is the direction of the field, not its magnitude, which determines direction of the FAC.

In all the analysis discussed in the following sections the input data are rotated to the local dipole system, the Swarm/DECS analysis is carried out there, and the output data are rotated back to the geographic coordinate system. EEJ flowing at the dip equator is one of the most prominent current systems at low magnetic latitudes, so it is important to ensure that it can be reproduced accurately. In our analysis there are no built-in assumptions about the location or latitudinal width of the EEJ. However, when using the 1D-DF SECS the current is assumed to flow in zonal direction of the local dipole system. This may not always coincide with the actual EEJ direction, but the difference should be small. Furthermore, the residual that is left from the 1D-DF SECS fitting is further analyzed using 2D-DF SECS, which make no assumption about the EEJ direction.

## Synthetic tests

We have created a number of synthetic test cases for assessing the performance of the Swarm/DECS analysis method at middle and low latitudes. These test cases are created with the Thermosphere Ionosphere Electrodynamics General Circulation Model (TIE-GCM, Roble et al. 1982, 1988; Richmond et al. 1992), with recent model updates described by Qian et al. (2014). TIE-GCM performs a 3D ionospheric current and conductance calculation, including wind dynamo currents, gravity and pressure-gradient driven currents and high-latitude field-aligned currents (Maute and Richmond 2017a; Maute and Richmond 2017b). Once the current systems are known, the magnetic disturbance can be calculated. This is done using spherical harmonic analysis and expressing the magnetic perturbation as a sum of toroidal and poloidal terms determined from the horizontal and vertical currents (see Eq. 8 in Maute and Richmond 2017a). The magnetic perturbations at the Swarm-A/C orbits are used as input in the Swarm/DECS analysis method, and the estimated currents are then compared to the simulated current based on TIE-GCM.

### TIE-GCM simulations and test cases

TIE-GCM is a physics-based model which self-consistently simulates the upper atmosphere. It solves the momentum, energy and continuity equations globally on a 3D grid for several neutral and ion species, taking into account influence of upward propagating atmospheric tides and external forcing from the magnetosphere via empirical ion convection and auroral particle precipitation models.

The ionospheric electrodynamic solver in TIE-GCM simulates all source terms, including the neutral wind dynamo, gravity and plasma pressure-gradient currents, and high-latitude magnetospheric energy input. Up to date information of the TIE-GCM setup can be found in the review articles by Qian et al. (2014) and Maute (2017).

We selected two representative days from an existing set of simulations (Maute 2017), one representing equinox conditions (23 September 2009) and the other representing solstice conditions (21 June 2009). Both days were geomagnetically quiet with $Kp \le 0+$ in September and $Kp \le 2+$ in June. This is appropriate for our testing purposes, as during quiet conditions the low and middle latitude current systems are in their most typical configuration.

The data we use consist of height-integrated horizontal currents in the E-region (TIE-GCM layers in the altitude range 80–222 km), the radial current at the upper boundary of the E-region current sheet and the magnetic field calculated at 442 km altitude, corresponding to the Swarm-A/C orbits. The currents are obtained in a geographical grid with $0.99^\circ \times 3^\circ$ spacing in latitude and longitude, respectively, while the magnetic perturbations are calculated in a $2^\circ \times 5^\circ$ geographical grid. This means that the synthetic magnetic data have a much lower spatial resolution than the real Swarm measurements (1.4$5^\circ$ longitude separation between the satellites, 1 Hz sampling corresponding to about 7.5 km along-track resolution). In order to avoid interpolation between the simulation data points, we use $5^\circ$ longitude separation between the Swarm-A/C satellites in the synthetic test cases and calculate the Swarm/DECS analysis results at the same points where the simulated currents are given. The orbital inclination of the Swarm-satellites is assumed to be 90$^\circ$, to match the TIE-CGM grid at fixed geographical longitudes.

The simulations give global snapshots of the ionospheric currents and magnetic field for every full hour for the two selected days. From this dataset we have selected 8 representative test cases, summarized in Table 1. They consist of 2 different geographical longitudes, 3 different local times (morning/noon/afternoon) and 2 seasons (equinox/solstice).

**Table 1:** Summary of our synthetic test cases

	T1	T2	T3	T4	T5	T6	T7	T8
Swarm-A long	$195^\circ$	$195^\circ$	$195^\circ$	$315^\circ$	$315^\circ$	$315^\circ$	$195^\circ$	$195^\circ$
Day	Sep-23	Sep-23	Sep-23	Sep-23	Sep-23	Sep-23	Jun-21	Jun-21
UT hour	18	22	02	10	15	19	18	21
LT hour	07	11	15	07	12	16	07	10

Orbit of Swarm-C is $5^\circ$ eastward of Swarm-A. Orbital altitude is 442 km and inclination is assumed to be $90^\circ$

At longitude $195^\circ$E the geomagnetic equator follows quite closely the ideal dipole equator, while around longitude $315^\circ$E the difference is large. The middle and low-latitude currents are small during night, increase quite rapidly in the morning and then decay during afternoon and disappear a few hours after sunset. Thus one could expect the currents to be relatively 1-dimensional (i.e., uniform in longitude) around noon, with larger longitudinal gradients in the morning and afternoon. The IHFAC are strongest during the solstices, flowing from the winter hemisphere to the summer hemisphere. In contrast, the EEJ is strongest during the equinoxes.

### Results

In order to avoid the assumption of strict anti-symmetry between the northern and southern hemispheres, the CF part of the Swarm/DECS analysis is done separately for each hemisphere. In contrast, the DF part of the analysis is done for both hemispheres at the same time, as it does not involve any symmetry assumptions. We present a detailed analysis of the test case T8 in Table 1. For the other test cases, we provide only a summary of the main results.

**Fig. 2:**
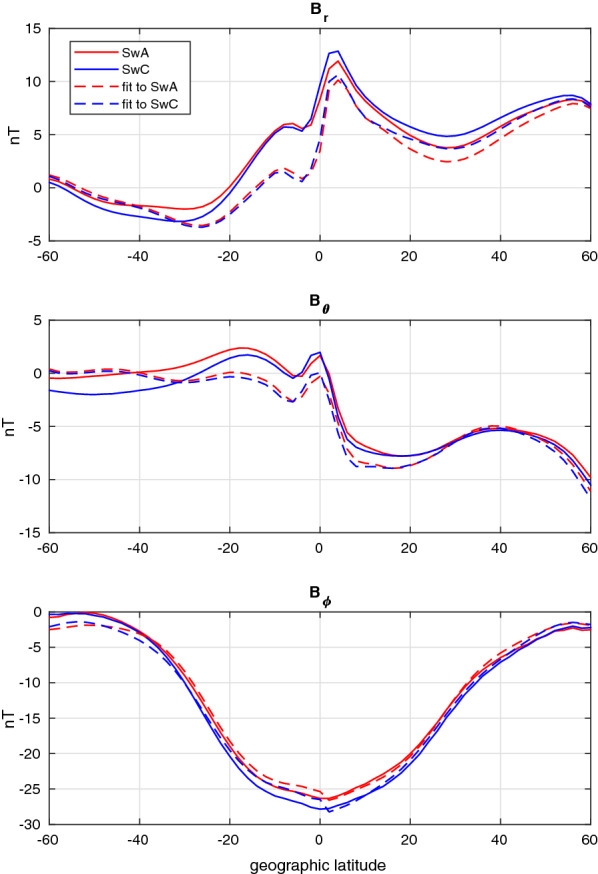
Test T8. Red and blue solid lines show the magnetic field components measured by Swarm-A/C, respectively, while the dashed lines show the fit from the Swarm/DECS analysis

Test T8 takes place at 10 local time close to the summer solstice, with Swarm-A flying along $195^\circ$ meridian. The synthetic measurements are shown in Fig. 2 together with the fit obtained in the Swarm/DECS analysis. Even though there are some offsets in the radial and southward components, the fit matches the measurement closely. It should be noted that the synthetic magnetic data include the total simulated 3D-current field, whereas the DECS consist of a current sheet and idealized IHFAC. Therefore the DECS may not be able to represent all the currents that are affecting the magnetic field (e.g., currents in the F-region). The relative difference in the magnetic field measured by the two satellites is rather small, indicating relatively small cross-track gradients in the currents. The symmetric shape of the eastward magnetic disturbance (bottom panel) indicates that IHFAC is indeed flowing from one hemisphere to the other.

**Fig. 3:**
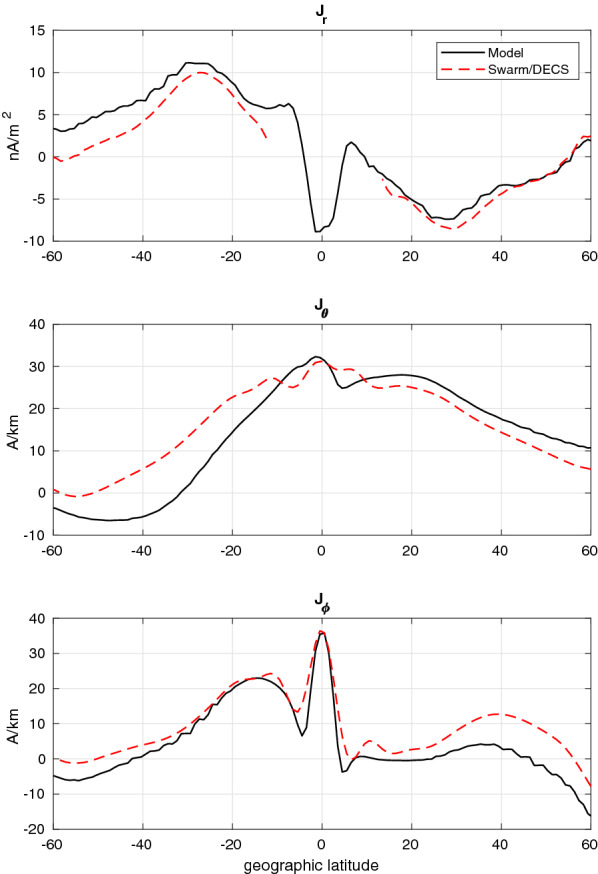
Test T8. Latitude profiles of the the current components between the Swarm-A/C satellites. Black solid lines show the simulated current, while red dashed lines show the Swarm/DECS analysis result. In the top panel upward current is positive

The latitudinal current profiles shown in Fig. 3 show a reasonable agreement between the model and analysis result. The radial current (top panel) at the top of the E-region current sheet is reproduced very well in the northern hemisphere, but in the southern hemisphere the current is slightly underestimated. There is indeed anti-symmetry between the hemispheres, with upward/downward currents around $+30^\circ / -30^\circ$ latitudes flowing from the winter to the summer hemisphere, as expected. Since the apexes of magnetic field lines close to the magnetic equator do not reach the satellite altitude, the CF DECS at these latitudes can not be fitted reliably and we leave this “exclusion zone” out when plotting the radial current density.

Also the southward current (middle panel) is reproduced rather well, although also here the southern hemisphere is more problematic. In contrast, in the eastward current component (bottom panel) the northern hemisphere has larger offsets, with the Swarm/DECS analysis slightly overestimating the current there. The EEJ flowing at the magnetic equator (very close to the geographical equator at this longitude) is reproduced very well, although in the Swarm/DECS result the width of the EEJ is slightly overestimated and the dips at either side of the EEJ are not quite deep enough.

**Fig. 4:**
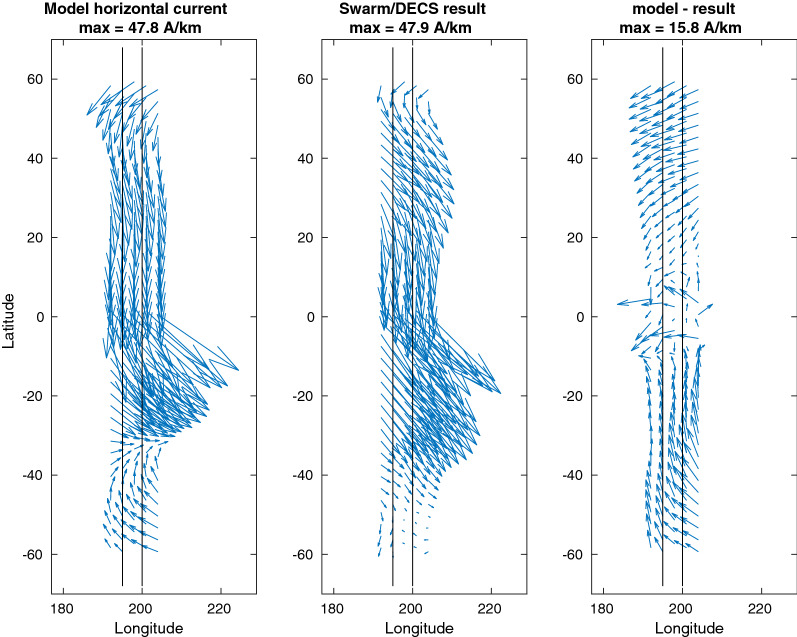
Test T8. Map of the E-region horizontal current. From left to right: the TIE-GCM model current, result from the Swarm/DESC analysis, and the difference between the two. Same scale is used in each panel. Swarm-A/C orbits are shown as black lines. Compare with the latitude profiles shown in Fig. 3

An important purpose of the Swarm/DECS analysis is to produce 2D latitude–longitude maps of the current in the E-region, around the Swarm-A/C trajectories and their magnetic footpoints. Figure 4 shows such a map about the horizontal current density. The two black lines show the Swarm-A/C orbits. Note that all the 3 panels have the same scale, and for clarity only every 3rd vector is plotted. The horizontal current map confirms that indeed the large-scale structure of the simulated current (left panel) is reproduced in the Swarm/DECS analysis (middle panel). However, there are also some differences, perhaps most notably in the high-latitude sides of the analysis area, poleward of about $\pm 40^\circ .$

**Fig. 5:**
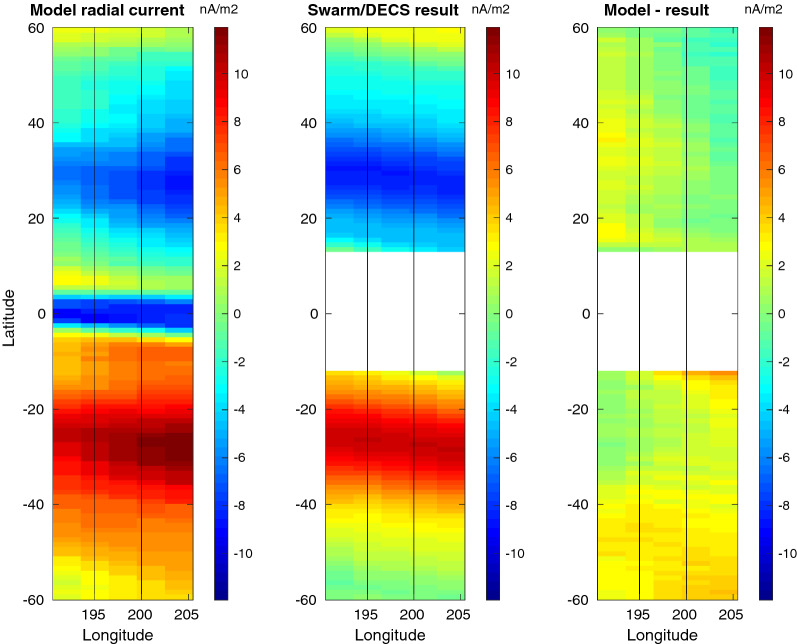
Test T8. Map of the radial current. From left to right: The TIE-GCM model current, result from the Swarm/DESC analysis, and difference between the two. Swarm-A/C orbits are shown as black lines. Compare with the latitude profiles shown in the top panel of Fig. 3

Figure 5 shows a similar map of the radial current, which in the simulation is the vertical current at the top of the ionospheric current sheet and in the Swarm/DECS result the divergence of the horizontal sheet current. It is also reproduced very well, although the Swarm/DECS result in the middle panel underestimates the longitudinal gradients. In the simulation there is a strip of intense downward current at the magnetic equator, which is not reproduced in the Swarm/DECS analysis due to the aforementioned exclusion zone.

To quantify the results, we calculate the mean absolute error (MAE) between the Swarm/DECS analysis results and the synthetic data. This is done separately for the horizontal and radial current densities, and also for the fit to the magnetic field data. For the horizontal sheet current $\vec {J}_\perp$ the absolute and relative MAE are defined as

8 $$\begin{aligned} MAE_A(\vec {J}_\perp ) = <|\vec {J}_{\perp ,\,model}-\vec {J}_{\perp ,\,result}|>, \end{aligned}$$

9 $$\begin{aligned} MAE_R(\vec {J}_\perp ) = 100 * \frac{MAE_A}{<|\vec {J}_{\perp ,\,model}|>}, \end{aligned}$$

respectively, and similarly for the magnetic field fit. For the radial current the error is calculated as for a one component vector. Here, | | is the usual length of a vector (L2-norm) and $<>$ means a spatial average over the chosen area. For the currents, we use a line of constant longitude between the satellites in the latitude range $\pm 60^\circ$, which corresponds to the latitude profiles shown in Fig. 3. For the magnetic field the error is calculated along the satellite orbits, combining the Swarm-A/C to the same MAE calculation. The absolute and relative errors are given in Table 2.

**Table 2:** Absolute (upper numbers) and relative (lower numbers) mean absolute errors in the 8 synthetic test cases, calculated at one longitude between the Swarm-A/C satellites

	T1	T2	T3	T4	T5	T6	T7	T8
$MAE(\vec {J}_\perp )$ [A/km]	6.6 42.3%	8.3 37.5%	8.3 58.7%	8.4 79.6%	13.1 47.4%	9.1 72.8%	9.5 56.6%	7.8 40.2%
$MAE(J_r)$ [nA/m$^2$]	1.6 70.8%	2.8 71.8%	1.0 22.7%	1.9 73.4%	1.7 50.0%	2.5 70.0%	0.8 29.3%	1.6 30.9%
$MAE(\Delta \vec {B})$ [nT]	1.2 18.3%	2.7 13.5%	6.4 52.0%	7.7 73.5%	8.1 44.0%	3.6 46.7%	1.7 24.9%	2.2 14.4%

Before drawing too many conclusions from Table 2, it should be noted that neither the absolute or relative MAE may fully reflect the general accuracy and usefulness of the solution. For example, in a situation where there are large (latitudinal) gradients in the current, a solution where the current profile is spatially shifted even by a small amount may result in a large MAE, despite giving otherwise good description of the situation. It is important to pay attention to the general structure and spatial pattern of the solution, in addition to the numerical MAE values.

From the definition in Eq. (9) it follows that a zero-solution has 100% relative MAE. The lowest relative MAE value in the horizontal current is 37.5% obtained in T2, with T8 having only slightly larger error. Taking a look at Fig. 3, we see that the solution is reasonably accurate and usable. The smallest relative MAE in the vertical current is 22.7% obtained in T3, followed by T7 and T8. Again the Swarm/DECS result in the top panel of Fig. 3 follows the simulated profile very well. Using the simple measure of adding up the relative MAE in the horizontal and vertical current, the best overall result is obtained in T8. In many test cases the relative MAE in the magnetic field fit are quite large, but the smallest values are obtained in T2 and T8, where also the horizontal current has a small error.

We have not shown the errors separately for the northward/eastward or CF/DF currents. That calculation reveals that the best results are obtained for the eastward component of the DF current. This current component includes the EEJ, which is indeed produced well in most of the test cases. This may be due to the fact that the EEJ produces a strong and reasonably localized magnetic signal. Also in those cases where there are some offsets in the absolute magnitude of the current, the relative magnitude of the EEJ, i.e., the difference compared to adjacent latitudes, is still reproduced quite well. This is encouraging, as the EEJ is an essential part of the low-latitude current systems and the focus of several studies.

According to Table 1, the test cases can be divided into 3 sequences, T1–T3, T4–T6 and T7–T8, so that in each sequence only the local time changes. The effect can be seen in the horizontal MAE (first row in Table 2). In the sequence T1–T3 the smallest error is achieved in T2, which takes place close to the local noon. Similarly, in sequences T4–T6 and T7–T8 the noon cases T5 and T8 have the smallest errors. This is an expected result: longitudinal gradients are usually smaller around noon, making the current system simpler. As can be noted in Figs. 4, 5, the Swarm/DECS analysis results do not seem to contain very many 2D structures, and this is true also in the other test cases.

In contrast, the vertical MAE (second row in Table 2) does not exhibit similar systematic variation with respect to the local time. Instead, if we consider T3 an outlier, we could conclude that the vertical error is smallest in the last two test cases. This may be related to the fact that the IHFAC are stronger during solstices, making them stand out more clearly in the magnetic disturbance.

The two most difficult test cases seem to be T4 and T6. Also T5 shows larger errors than the other two noon tests T2 and T8, at least in the horizontal current. This may be caused by tests T4–T6 taking place around longitude $315^\circ$E, where the main field is strongly non-dipolar, in contrast to the longitude $195^\circ$E where the other test are located.

All these features indicate that in middle and low-latitude applications the Swarm/DECS method works more reliably in some conditions than in others. The best results would probably be achieved around local noon close to the summer or winter solstice at a location where the main field is close to a dipole. However, even in cases where the MAE are larger, many features (like the EEJ and large-scale FACs) are still in good qualitative agreement with the synthetic data.

Finally, we note that the relative errors tend to be much larger than those in the high-latitude Swarm/SECS method (e.g., Amm et al. 2015; Vanhamäki et al. 2020). We speculate that this may be at least partly due to more versatile physics of the ionospheric currents at lower latitudes. At high latitudes, the horizontal currents are concentrated to a narrow altitude range in the E-region, forming the current sheet. At satellite altitude the currents are almost perfectly field-aligned. The magnetic disturbances produced by the E-region horizontal currents and FACs are typically of the order of 100 nT or more, overwhelming magnetic signals from any other F-currents that might be present. In contrast, in our middle and low-latitudes test cases the magnetic disturbances rarely exceed 30 nT. In these conditions, even weak currents that may be present in the ionospheric F-region, above the nominal E-region current sheet, may have noticeable effect on the measured magnetic disturbance. This includes for example the pressure-gradient and gravity currents (Alken et al. 2016) and the F-region dynamo current (Maute and Richmond 2017b).

## Application to Swarm data

In addition to the synthetic tests, we have also applied the Swarm/DECS analysis method to 3 equatorial passes made by the Swarm-A/C satellites. These 3 events, called E1–E3, are summarized in Table 3. They have been previously analyzed by Alken et al. (2017). Events E1 and E2 take place in January 2015, close to the winter solstice, while E3 is from March 2016 and represents equinox conditions. The chosen events occur during daytime, covering local times between 10:32 and 14:21, when the equatorial electrojet and IHFAC are expected to be strong. All events were magnetically quiet, with the Kp index between 1 and 2-.

**Table 3:** Summary of the selected events.

	Longitude	Date	UT	LT	Kp
E1	$142^\circ$	01-Jan-2015	04:55	14:21	1
E2	$314^\circ$	18-Jan-2015	15:53	12:39	1
E3	$349^\circ$	18-Mar-2016	11:22	10:32	2-

The longitude, UT and local time correspond to Swarm-A crossing the geographical equator

The analysis itself is carried out the same way as in the synthetic test cases. We use the 1 Hz Swarm magnetic field data (product baseline 0505) and subtract the background field taken from the CHAOS-6 model (Finlay et al. 2016). The parameters used in grid generation and regularizing the matrix inversions with truncated singular value decomposition were the same as in the synthetic test cases. We analyzed the Swarm data between geographic latitudes $\pm 60^\circ$, but the results are shown only in the interval $\pm 40^\circ$ in order to avoid auroral effects.

Now the error can be calculated only for the magnetic field fit. The horizontal current can be qualitatively compared with the results obtained by Alken et al. (2017), although their analysis was limited to the DF part of the current. The radial current derived with the Swarm/DECS method are compared to the Level-2 dual-satellite data product (product baseline 0301) estimated using the quad method as described by Ritter et al. (2013). For this comparison the FAC from the quad method is first mapped down to the E-region current sheet along the magnetic field lines, and is then converted to radial current. The magnetic footpoint locations are calculated with the AACGM coordinate conversion (Shepherd 2014).

**Fig. 6:**
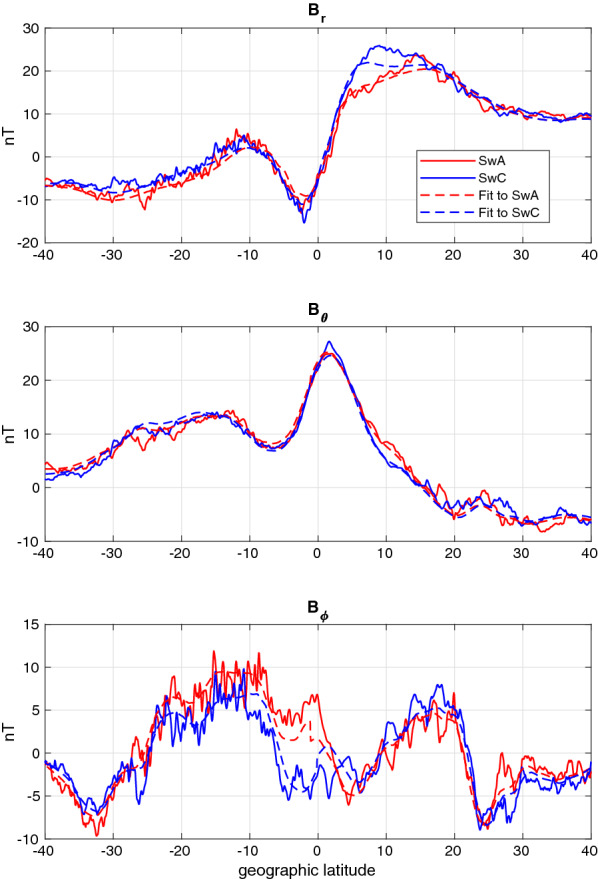
Event E2. Red and blue solid lines show the magnetic field components measured by Swarm-A/C, respectively, while the dashed lines show the fit from the Swarm/DECS analysis

We show detailed results only for one event, namely E2. It takes place about a month after the winter solstice, when the IHFAC is expected to be stronger and the EEJ weaker than during equinoxes. Swarm-A crosses the geographic equator at longitude $314^\circ$ around 12:39 local time. Figure 6 shows the measured magnetic field components together with the Swarm/DECS fit. The magnetic field data contain much more small-scale structures than in the synthetic test cases (which had $2^\circ$ latitude resolution), but the fit is good.

**Fig. 7:**
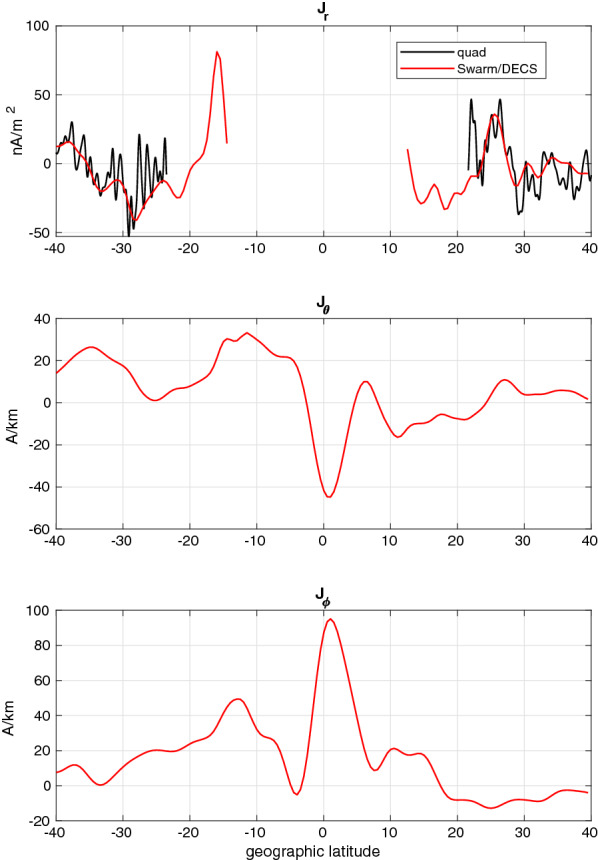
Event E2. Latitude profiles of the the current components between the Swarm-A/C satellites obtained from the Swarm/DECS analysis. Black solid line in the upper panel shows the radial current from the quad method (Ritter et al. 2013)

**Fig. 8:**
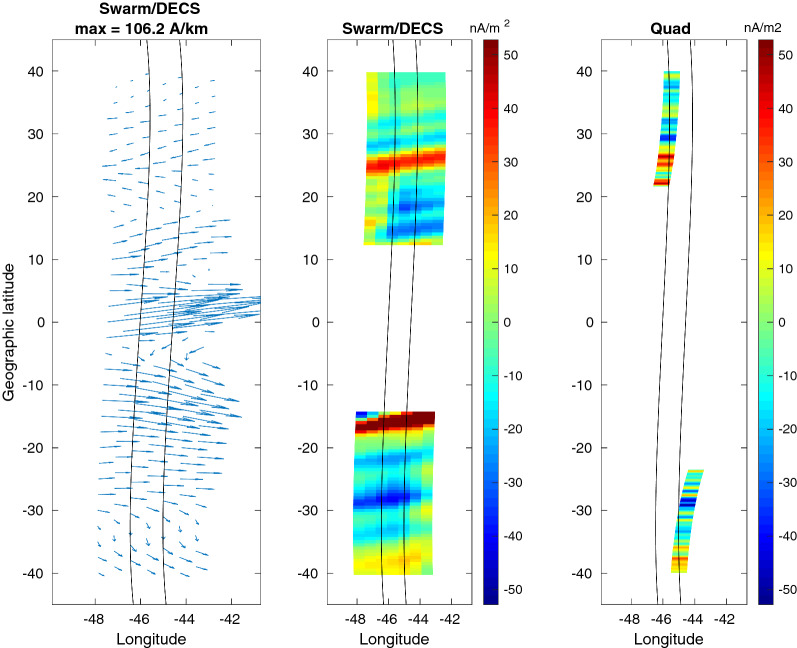
Event E2. Maps of the E-region horizontal and radial currents. From left to right: the horizontal current from Swarm/DECS analysis the radial current from the Swarm/DECS analysis and the radial current from the quad method (Ritter et al. 2013). Swarm-A/C orbits are shown as black lines. Compare with the latitude profiles shown in Fig. 7

The resulting current profiles are shown in Fig. 7 and the current maps in Fig. 8. The correspondence between the quad and Swarm/DECS radial currents is good, although the quad method is able to estimate the current at clearly better spatial resolution. It can be noted that the Swarm/DECS result extends closer to the magnetic equator than the quad result, which is limited by the inclination of the magnetic field. We note that the direction of the radial current around $20^\circ - 30^\circ$ latitude in each hemisphere is as expected from previous results (e.g., Olsen 1997; Lühr et al. 2015), that is mostly upward in the northern hemisphere (winter) and mostly downward in the southern hemisphere (summer). However, according to the Swarm/DECS results the current direction is reversed closer to the equator. The radial current map shows some 2D features, which provide an interesting context to the quad current. In the leftmost plot the quad current is displaced from the satellite tracks due to the magnetic mapping: the tracks show the satellite location, but the current is mapped to the E-region.

The horizontal current is dominated by the EEJ, which is slightly tilted northwards and is located around latitude $2^\circ$. When compared to the result shown in Figure 8 of Alken et al. (2017), it is clear that apart from the EEJ the two results are somewhat different. The current obtained by Alken et al. (2017) has a large poleward component at mid-latitudes in both hemispheres, whereas our result shows westward or southwestward currents at northern mid-latitudes. We can only speculate about the reason, but there seem to be 5–10 nT offsets in the magnetic field components that we used in the analysis (Fig. 6) and those used by Alken et al. (2017), which may contribute to the differences in the estimated currents.

The three events with real Swarm data give a bit different impression of the Swarm/DECS method than the 8 synthetic test cases discussed previously. The fitted magnetic field is very close to the Swarm measurements, with absolute errors of 2.4 nT, 2.2 nT and 1.5 nT in the events E1, E2 and E3, respectively. The corresponding relative errors are 14.4%, 14.4% and 11.3%. These are comparable to or better than the best fits obtained in the synthetic test cases (see Table 2). This means that the DF and CF DECS basis functions have no problems in representing the actual magnetic field data and the resulting current profiles should be at least as accurate as the synthetic results shown in Figs. 3, 4,5. Indeed, the current profiles (like Fig. 7) as well as the current maps (Fig. 8) seem to be very realistic.

The quad method used in the level-2 data product and the Swarm/DECS method give very similar results for the large-scale radial current, but the spatial (i.e., latitudinal) resolution in the Swarm/DECS result is not as good. This may be partly due to the analysis parameters that we used: they were originally selected to work with the synthetic test cases, which have very coarse resolution compared to the real data. However, the Swarm/DECS method has clearly a better latitude coverage than the quad method, extending closer to the magnetic equator.

The horizontal currents show a clear EEJ in all the events. The locations and directions of the EEJ agree very well with the location of the geomagnetic equator and the results obtained by Alken et al. (2017). Quantitative comparisons with Alken et al. (2017) are difficult, but it is clear that there are some differences, especially at mid-latitudes. But even in qualitative comparisons it should be noted that the main motivation of Alken et al. (2017) was to estimate the global (or at least very large scale) current system, so their fit to the magnetic data is rather approximate.

## Summary and conclusions

We have modified and developed the SECS analysis method so that it can be used also at middle and low latitudes. This required redefinition of the 1D and 2D-curl-free basis functions, as they are connected to the FAC. We redefined them so that the FAC flows from one hemisphere to the other along dipole field lines. Hence, we call them DECS (dipolar elementary current systems). The divergence-free basis functions are not connected to the FAC, so they do not require changes.

Our main application of the new analysis method is to estimate ionospheric currents at middle and low latitudes from magnetic data provided by the Swarm-A/C spacecrafts. As the Earth’s magnetic field deviates from an ideal dipole, the Swarm/DECS analysis is performed in a local dipole coordinate system, where the field is as close to an ideal as possible. To allow for hemispheric differences, the CF part of the analysis is done separately for each hemisphere.

We note that instead of the local dipole system, a better approximation would be to use IGRF or similar magnetic field model to calculate the IHFAC flow direction. In principle this would not change the procedure very much, as the Biot–Savart integral along the field line is calculated numerically in any case. However, one of our goals is to have a method that is applicable at a wide latitude range, and in practice numerical field-line tracing would be difficult at high latitudes. Moreover, in the local dipole approximation the 1D-CF SECS have an analytical expression, which is very fast to evaluate. In a more realistic field geometry the 1D-CF SECS must also be calculated numerically.

In order to assess the performance of the Swarm/DECS method in different geophysical situations we prepared 8 synthetic test cases. In general the Swarm/DECS results give a good qualitative and often also quantitative description of the currents, although there are also some outliers. The absolute and relative errors given in Table 2 indicate that some geophysical conditions (e.g., local noon, solstice and dipole-like main field) lead to better estimates of the current system.

We applied the Swarm/DECS method to 3 events using magnetic field data from the Swarm-A/C spacecrafts. The radial currents obtained with the Swarm/DECS method are in good agreement with the quad method (Ritter et al. 2013) used for the Swarm data product. Also in the horizontal current and especially in the EEJ there is a good qualitative correspondence between the Swarm/DECS analysis and results presented by Alken et al. (2017) for the same events.

The main advantage of the Swarm/DECS method is the 2D view of the ionospheric current system, which greatly expands the view offered by the present Swarm data products. This becomes most evident in the 3 event studies, one of which is illustrated in Fig. 8. The important question is of course how reliable these current maps are, especially outside the satellite tracks. Unfortunately we do not have a clear answer at the moment. The maps look realistic, but the lack of 2D structures in the synthetic test cases raises some concerns.

Based on these results, we can conclude that the low-latitude Swarm/DECS method works in practice. It is a very promising research tool, but additional testing, using both synthetic data and real measurements, is needed before wide-spread applications. The synthetic test models should have similar spatial resolution and variation as the actual Swarm data. Overflights of ground-based magnetometer networks and future conjunctions between the Swarm-A/C pair and Swarm-B (expected during year 2021–2022) may offer good validation opportunities.

## Data Availability

ESA is acknowledged for providing the Swarm data, available at ftp://swarm-diss.eo.esa.int. The Technical University of Denmark (DTU) is acknowledged for providing the CHAOS-6 magnetic field model, available at http://www.spacecenter.dk/files/magnetic-models/CHAOS-6/. The TIE-GCM simulation runs used in the synthetic test cases are available on reasonable request from HV or AM. The Swarm/DECS analysis code is available on reasonable request from HV.

## Appendix 1

The magnetic field of the 2D-CF DECS defined in Eq. (4) can be calculated in two parts: the magnetic field of the horizontal ionospheric currents placed at the northern and southern hemispheres, plus the field of the dipolar line current flowing between the hemispheres.

## Part 1: spherical 2D-CF SECSs without line currents

The horizontal current can be obtained by removing the magnetic field of a semi-infinite radial line current flowing to/from the SECS pole from the expression of the magnetic field of a 2D-CF SECS (Eq. (2.15) of Vanhamäki and Juusola 2020). We note that in addition to the semi-infinite line current, the 2D-CF SECS (as illustrated in figure 2.1 of Vanhamäki and Juusola 2020) includes also a uniform FAC distribution spread all over the sphere. This uniform FAC is not removed from the system, but it cancels out in the final construction, as discussed below.

The field of a semi-infinite line current of amplitude $I_0$ flowing along $\theta '=0$ from $r=R_I$ to infinity is

10 $$\begin{aligned} \vec {B}_{semi}(r,\theta ^{\prime},\phi ^{\prime}) = \frac{-\mu _0 I_0}{4 \pi } \, \frac{\vec {e}_{\phi ^{\prime}}}{r\sin \theta ^{\prime}} \, \left( \frac{R_I - r\cos \theta '}{\sqrt{r^2+R_I^2-2rR_I\cos \theta ^{\prime}}} - 1 \right) \end{aligned}.$$

The primed coordinate system is centered at the SECS pole, which is in the (geomagnetic) location $\vec {r}^{\,el} = (R_I, \theta _0, \phi _0)$. The magnetic field of the CF horizontal current and uniform FAC is

11 $$\begin{aligned} \vec {B}_{1}(r,\theta ^{\prime},\phi ^{\prime})= & \, \vec {B}^{ 2DCF } + \vec {B}_{semi} \\= & \, \frac{-\mu _0 I_0}{4\pi } \, \frac{\vec {e}_{\phi ^{\prime}}}{r\sin \theta ^{\prime}} \, \left\{ \begin{array}{ll} \frac{s-\cos \theta ^{\prime}}{\sqrt{1+s^2-2s\cos \theta ^{\prime}}} + \cos \theta ^{\prime}, &{} r>R_I, \\ \frac{1-s\cos \theta '}{\sqrt{1+s^2-2s\cos \theta '}} -1, &{} r<R_I. \end{array} \right. \nonumber \end{aligned}$$

Here $s=min(r,R_I)/max(r,R_I)$ and $\vec {B}^{ 2DCF }$ is the field of the 2D-CF SECS.

As mentioned above, the current system consistent with $\vec {B}_{1}$ in the above equation contains a uniform FAC distribution spread all over the sphere. However, in the 2D-CF DECS we place two such current systems with opposite directions at conjugate points $\theta _0$ and $\pi -\theta _0$, as in Eqs. (4-5). When calculating their sum, the uniform FACs cancel each other.

## Part 2: dipolar line current

The field line starts from the southern 2D-CF SECS pole at (geomagnetic) location $(R_I,\pi -\theta _0,\phi _0)$. Assuming $0<\theta _0<\pi /2$ this correspond to the convention that positive amplitude of the 2D-CF DECS means current flow from the southern to the northern hemisphere. Points $\vec {r}_D$ in this field line have Cartesian coordinates

12 $$\begin{aligned} x_D= & \, L \sin ^3\theta _D \cos \phi _0 \nonumber \\ y_D= & \, L \sin ^3\theta _D \sin \phi _0 \\ z_D= & \, L \sin ^2\theta _D \cos \theta _D, \nonumber \end{aligned}$$

where $L = R_I / \sin ^2\theta _0$, and $\theta _0 \le \theta _D \le \pi -\theta _0$. Cartesian components of the line element $\vec {dl}$ along the dipole field line are

13 $$\begin{aligned} dl_x= & \, 3L\cos \theta _D \sin ^2\theta _D \cos \phi _0 \, \mathrm {d\theta _D}\nonumber \\ dl_y= & \, 3L\cos \theta _D \sin ^2\theta _D \sin \phi _0 \, \mathrm {d\theta _D} \\ dl_z= & \, -L (1-3\cos ^2\theta _D) \sin \theta _D \, \mathrm {d\theta _D} \nonumber \end{aligned}$$

The magnetic field is obtained by Biot–Savart integral,

14 $$\begin{aligned} \vec {B}_{2}(\vec {r}) = \frac{\mu _0 I_0}{4\pi } \int _{\pi -\theta _0}^{\theta _0} \frac{\vec {dl} \times (\vec {r}_D-\vec {r})}{|\vec {r}_D-\vec {r}|^3}. \end{aligned}$$

We have not been able to calculate this analytically. Instead we calculate the integral numerically by dividing the co-latitude range into *N* uniform segments of size $\Delta \theta _D$. This results in

15 $$\begin{aligned} \vec {B}_{2}(\vec {r}) \approx \frac{\mu _0 I_0}{4\pi } \sum _{i=1}^{N} \frac{\vec {dl}_i \times (\vec {r}_{D,i}-\vec {r})}{|\vec {r}_{D,i}-\vec {r}|^3}. \end{aligned}$$

Cartesian components of the vectors $\vec {dl}_i$ and $\vec {r}_{D,i}$ are calculated using the above expressions, at co-latitudes $\pi - \theta _0 + (i-0.5)\Delta \theta _D$ along the dipole field line (note that $\Delta \theta _D < 0$).

## Abbreviations

1D: 1-Dimensional
2D: 2-Dimensional
3D: 3-Dimensional
CF: Curl-free
DECS: Dipolar elementary current systems
DF: Divergence-free
E1–E3: Events 1–3
EEJ: Equatorial electrojet
ESA: European Space Agency
FAC: Field-aligned current
IHFAC: Interhemispheric field-aligned current
LEO: Low-Earth-orbiting
LT: Local time
MAE: Mean absolute error
SECS: Spherical elementary current systems
Sq: Solar-quiet
T1–T8: Tests 1–8
TIE-GCM: Thermosphere ionosphere electrodynamics general circulation model
UT: Universal time
